# A Case

**Published:** 1879-09

**Authors:** J. A. Goldsberry

**Affiliations:** Annapolis, Ind.


### NOTES FROM PRIVATE PRACTICE.

### ARTICLE VII.

*A Case.*

On the morning of April 7, 1878, I was called to meet in consultation my friend, Dr. I. H. Gillum, in a case in whose subsequent clinical history we became very much interested, not so much because of anything specially *new* in the symptoms, as from the difficulty in arriving at a clear and satisfactory diagnosis.

The patient was a married lady, the wife of a farmer, about sixty-five years of age, of a phlegmatic temperament, spare habit, and was free from any hereditary vice.

The doctor informed me that she had been complaining about a month with pain in the left ear; that while he was not entirely satisfied as to the nature of the trouble, he had been disposed to regard it as an obstinate form of neuralgia, and had treated it accordingly; latterly, however, he had feared abscess somewhere among the cranial bones.

A careful examination of the case revealed no apparent lesion anywhere. The tongue was but slightly furred, the appetite was fair, there was no febrile excitement, nor had there been at any time previous. There was no acceleration of the pulse, no disturbance of the circulation, nor was there in the region of pain, a particle of swelling or soreness. An examination through the speculum of the external meatus gave no sign of disease, the walls of that canal, together with the membrana tympani, presenting, apparently, a normal appearance. The mouth and pharynx were also examined with like results. The pain in the ear was deeply seated, of a dull, grinding character, was persistent, and generally more severe at night than through the day. It might also be proper to state that the patient complained of a slight irritation and weakness of the left eye, and numbness of the muscles of the corresponding side of the face.

Considering the fact that there seemed to be a total absence of all inflammatory excitement, and that the ordinary symptoms of abscess were not well marked, I was inclined to concur with the doctor in his diagnosis of the case; that is, that the affection probably belonged to the neuroses, and therefore was purely functional. While, up to this time, the case had received such treatment, both constitutional and local, as seemed indicated, it was decided that essentially the same line of therapeutic measures should be continued, for awhile at least.

During the following three or four weeks the anti-neuralgic treatment was kept up vigorously and persistently. Quinine was given to cinchonism; strychnia, iron, arsenious acid, aconite, belladonna and opium were all employed, either in combination or separately; vesication with iodine and blistering cerate behind and in front of the ear, was had recourse to without stint; but all without the least beneficial effect to the patient, save to afford, now and then, a little temporary relief from pain. It was now two months since the inception of her illness, and there had quite recently developed some new features in the case.

These were of such a character as to force the abandonment of the functional theory. A fullness was observed just in front of, and below, the left ear; indeed the swelling seemed to follow up and embrace the entire paroted gland. There was also a partial closure of the auditory meatus. A copious and profuse salivation with a feeling of fullness of the left side of the face, and a burning, smarting pain of the mouth of the corresponding side, were prominent symptoms. The mucous membrane of the mouth and pharynx of the affected side was irritable and highly congested, without, however, any abrasion of its surface. At the upper part of the pharynx, at its junction with the soft palate, a little to the left of the median line, was noticed a rounded or globular enlargement, which was firm and unyielding to the touch, and almost entirely devoid of sensibility.

The patient was evidently wearing; the appetite had become seriously impaired, and the loss of flesh and strength was marked and decided. During the next two months, while there was observed no new feature in the case, there was certainly an aggravation of all the symptoms enumerated, with a steady and rapid decline in the general health.

The paroted gland continued to enlarge, was comparatively painless, and as hard almost as bone. The growth at the upper margin of the pharynx increased in size, so much so, indeed, that we could not well divest ourselves of the belief that within this tumor, or in its immediate vicinity, there must be a collection of pus. With the view of confirming or otherwise setting at rest this question, we repeatedly, from time to time, carefully examined it with an exploring needle; also passed to its entire depth a fine-pointed, narrow-bladed bistoury, but always with negative results.

The saliva still continued to pour from the mouth in incredible quantity, was pungent and acrid, burning and smarting the mucous surfaces, and gave off a very offensive, nauseous odor. The left eye was inflamed and painful, with vision almost totally obscured ; the auditory meatus, from which exuded a thin, serum-like fluid, was gradually closing, and the functions of the ear were very nearly destroyed. On carefully examining the meatus with a probe, it was passed without difficulty, not only to the bottom of this canal, but through the membrana tympani into the middle ear, thence through this cavity and beyond, apparently, along the entire track of the eustachian tube.

The *numbness* of the left cheek, already alluded to, had developed into almost complete anæsthesia, and this loss of sensibility was not confined to the facial muscles, but embraced, so far as observed, all of the muscles supplied by the fifth nerve, as these could be pinched or pricked without giving rise to the least sensation. At this time, the sixteenth week of her illness, the patient became suddenly unconscious. The loss of consciousness was soon followed by slow and stertorous breathing and coma, more or less profound. In short, the attack was purely apoplectic in its nature, accompanied by a train of symptomatic phenomena such as always follow compression of the cerebral substance.

This condition lasted five or six hours, when there was a gradual return to consciousness ; but it was found that the whole of the *left* side had become hemiplegic ; there was total blindness of the left eye, the sense of hearing in the left ear was completely abolished, and the facial muscles of the affected side were so drawn to the opposite side as to give to the physiognomy a most unnatural and hideous expression.

The paralysis, so far as motility was concerned, seemed complete, and continued until the close of life. The condition of the patient was now indeed pitiable ; the paralysis of the muscles concerned in deglutition rendered alimentation by the mouth imperfect and difficult. The integument was corrugated and shriveled, and, in color, not unlike that of the cadaver ; the emaciation, which had been marked for two months, had, it appeared, reached the utmost limit, and to add still more to the distressing features of the case, bed sores were rapidly forming over the iliac projections, the great trochanters, and indeed on almost every part of the body subjected to pressure.

The patient continued to fail rapidly, and died from exhaustion four weeks from the time of the apoplectic seizure, and about five months from the first symptoms of illness. Death was peaceful and quiet, wholly free from convulsions of any character.

From the above history of this case, though briefly and imperfectly given, enough has, I trust, been said, to make it evident that whatever may have been the nature of the lesion, and wherever may have been its primary seat, sooner or later it became intra-cranial, involving the cerebral structure in such a way, and to such an extent as to greatly hasten the fatal issue, and render death inevitable.

What was the matter, and where was the starting point of the disease? Was it primarily an abscess of the mastoid cells, or was it scirrhus, affecting some one or more of the cranial bones? If neither of these, then what was it?

Again, observe that the paralysis occurred on the *left* or diseased side. How are we to reconcile this fact with the accepted theory that hemiplegia, when it takes place, is always the result of cerebral lesion affecting the *opposite* side to the one paralyzed?

ANNAPOLIS, IND. J. A. GOLDSBERRY, M.D.

